# National Guard or Continental Army

**Published:** 1916-03

**Authors:** 


### National Guard or Continental Army.

Modesty forbids more than brief allusion to the resignation of Secretary-of-War Garrison, after our declaration in the previous issue in favor of the development of the National Guard. But, seriously, is not the development of a national policy by the free expression of opinion throughout the country, when no one individual or association of individuals can claim proprietorship in an idea or influence to carry it into execution, the more democratic way, and the one most likely to secure ultimately, the wisest decision? Note that the editorial referred to was not a single personal opinion. It was virtually a symposium, representing general patriotism, the aspiration of medical men untrained in military service to secure efficiency for such service, if necessary, and the experience of both medical, staff and line officers of long service in the National Guard. By all of these factors, personal judgement was subordinated to the desire to secure the best final decision. The issue has been simplified by what appears to be the decision that the main defense of our country, in the numeric sense, must rest with the National Guard. This organization must be increased, perfected, and nationalized. Probably through its instrumentality, in some way, the partial instruction of the ultimate militia of the country must be brought about, in order to provide sufficient material, not quite in the raw state, for an emergent increase of military forces. We are especially interested in the problem of such instruction for a potential military staff of adequate numbers. We invite further discussion of this subject from our readers.

